# In-plane anisotropic third-harmonic generation from germanium arsenide thin flakes

**DOI:** 10.1038/s41598-020-71244-y

**Published:** 2020-08-31

**Authors:** Huseyin Sar, Jie Gao, Xiaodong Yang

**Affiliations:** Department of Mechanical and Aerospace Engineering, Missouri University of Science and Technology, Rolla, MO 65409 USA

**Keywords:** Nonlinear optics, Two-dimensional materials

## Abstract

A newly introduced two-dimensional (2D) layered germanium arsenide (GeAs) has attracted growing interest due to its promising highly in-plane anisotropic crystal structure and electronic properties for photonic and optoelectronic applications. The potential of 2D layered GeAs for many applications such as anisotropic photodetection, electronics, superconductivity and thermoelectricity is being investigated in recent studies. However, the intrinsic nonlinear optical properties of 2D layered GeAs have not been explored yet. Here, thickness- and incident polarization-dependent in-plane anisotropic third-harmonic generation (THG) from the mechanically exfoliated thin GeAs flakes is reported. Furthermore, the effect of the flake thickness on the THG conversion efficiency is shown to find the optimal thickness range for high conversion efficiency. The polarization state of the emitted THG signal is also analyzed by measuring the Stokes parameters with different polarization states of the pump beam to demonstrate the capability of controlling the intensity and polarization of TH emission. Our results will create new opportunities for advancing anisotropic optical devices used for future photonic integration, optical communication and optical information processing.

## Introduction

Nonlinear optical materials play a progressively significant role on the development of important photonic devices, for example, ultrafast lasers^1^, soliton generators^2^, optical modulators^3^ and optical multiplexers^4^, which are extensively used in many applications of medicine, industry, communication and imaging^4,5^. Although the traditional bulk nonlinear optical materials such as beta barium borate (β-BaB_2_O_4_)^6^ and lithium niobite (LiNbO_3_)^3^ have been widely used, these materials are greatly limited by low conversion efficiency, low nonlinear optical susceptibility and bulky crystal size. It is crucially important to search for nonlinear optical materials with both high conversion efficiency and compact crystal size, which can be integrated into the future nanoscale photonic and quantum chips^7–9^. Within this scope, 2D materials have been the most promising candidates to fulfill these demands^10^. The emerging 2D layered materials are currently at the center of intensive research efforts owing to their superior physical properties such as high carrier mobility^11,12^, broadband optical response^13^, large Young’s modulus^14,15^ and high thermal conductivity^16^. In addition, the broad optical spectral range covered by 2D materials make them very promising for photonic and optoelectronic applications^17^. Besides the linear optical properties, 2D materials exhibit strong optical nonlinearities which endows them as promising nonlinear optical materials^10,18,19^ which have been used for saturable absorption (SA)^20^, second-harmonic generation (SHG)^21^, THG^22,23^, self-phase modulation^24,25^, nonlinear Kerr effect^26,27^ and four-wave mixing (FWM)^28^. Beyond graphene, 2D transition metal dichalcogenides (TMDs) such as MoS_2_^21,29–31^ and WSe_2_^32,33^, hexagonal boron nitride (h-BN)^21^, and other kinds of materials such as ReS_2_^34,35^ and GaSe^36^ have shown strong SHG and THG responses.

Furthermore, the in-plane anisotropy in 2D materials due to the reduced crystal symmetry is envisioned as another degree of freedom to manipulate the electrical, optical, thermal or mechanical properties. Several kinds of 2D materials with highly in-plane anisotropy such as black phosphorus (BP)^37^, GeSe^38^, ReS_2_^35^ and SnSe_2_^39^ have attracted growing attention. The combination of highly in-plane optical anisotropy and other physical properties coming from the layered nature of 2D materials provides new opportunities for making novel photonic devices in unique applications such as polarization sensitive photodetectors^40,41^, polarization sensors^42^, digital inverters^43^ and anisotropic memorizers^44^. As a widely studied anisotropic 2D materials, BP exhibits highly anisotropic nonlinear optical properties in the third-order nonlinear processes of THG^45^, SA^46^ and FWM^10^, but the lack of sufficient stability in ambient conditions further limits its potential for practical applications^47^. Therefore, different types of low-symmetry 2D materials with high in-plane anisotropic nonlinear optical properties and high ambient stability are being explored. In that context, a layered group-IV monochalcogenide of germanium selenide (GeSe), which is stable under ambient conditions, has been recently studied to show strong anisotropic nonlinear optical response in THG process with high conversion efficiency due to the low in-plane lattice symmetry^48^. GeSe has an orthorhombic crystal structure belonging to the Pnma space group with the puckered honeycomb lattice similar to the crystal structure of BP^49^. It is known that the nonlinear optical properties of materials are highly sensitive to the crystal structures and lattice symmetries^50,51^. Within this framework, unlike BP and GeSe, one layered group IV-V semiconductor of germanium arsenide (GeAs)^52^, which exhibits a monoclinic crystal structure belonging to the C2/m space group and is ambient stable, can be considered to show high in-plane anisotropic nonlinear responses. Recently, 2D GeAs has attracted increasing interest with its promising highly in-plane anisotropic crystal structure^53^ and high carrier mobility^54^ for applications in photodetection, superconductivity and thermoelectricity^54–57^. However, the intrinsic nonlinear optical properties of 2D layered GeAs have not been investigated yet.

In this work, we demonstrate the strong in-plane anisotropic THG in exfoliated GeAs flakes with different thicknesses. It is shown that the third-order nonlinear optical response is highly anisotropic with respect to the incident linear polarization of pump beam according to both theoretical and experimental analysis. The effect of the flake thickness on the THG conversion efficiency is also investigated and an optimal thickness range of 40–120 nm for achieving high conversion efficiency is revealed. In addition, the anisotropy ratio for the GeAs flakes with different thicknesses is determined from the polarization-dependent THG measurements. Moreover, the polarization state of the emitted THG signal is also analyzed by measuring the Stokes parameters for linearly and elliptically polarized incident pump beam. It is shown that the intensity and polarization state of THG signal can be manipulated by changing the incident polarization of pump beam. Our results will provide new opportunities for building anisotropic nonlinear optical devices used for future photonic integrated circuits, optical communication and optical information processing.

## Results

### Crystal orientation determination of exfoliated GeAs flakes

**Figure 1:**
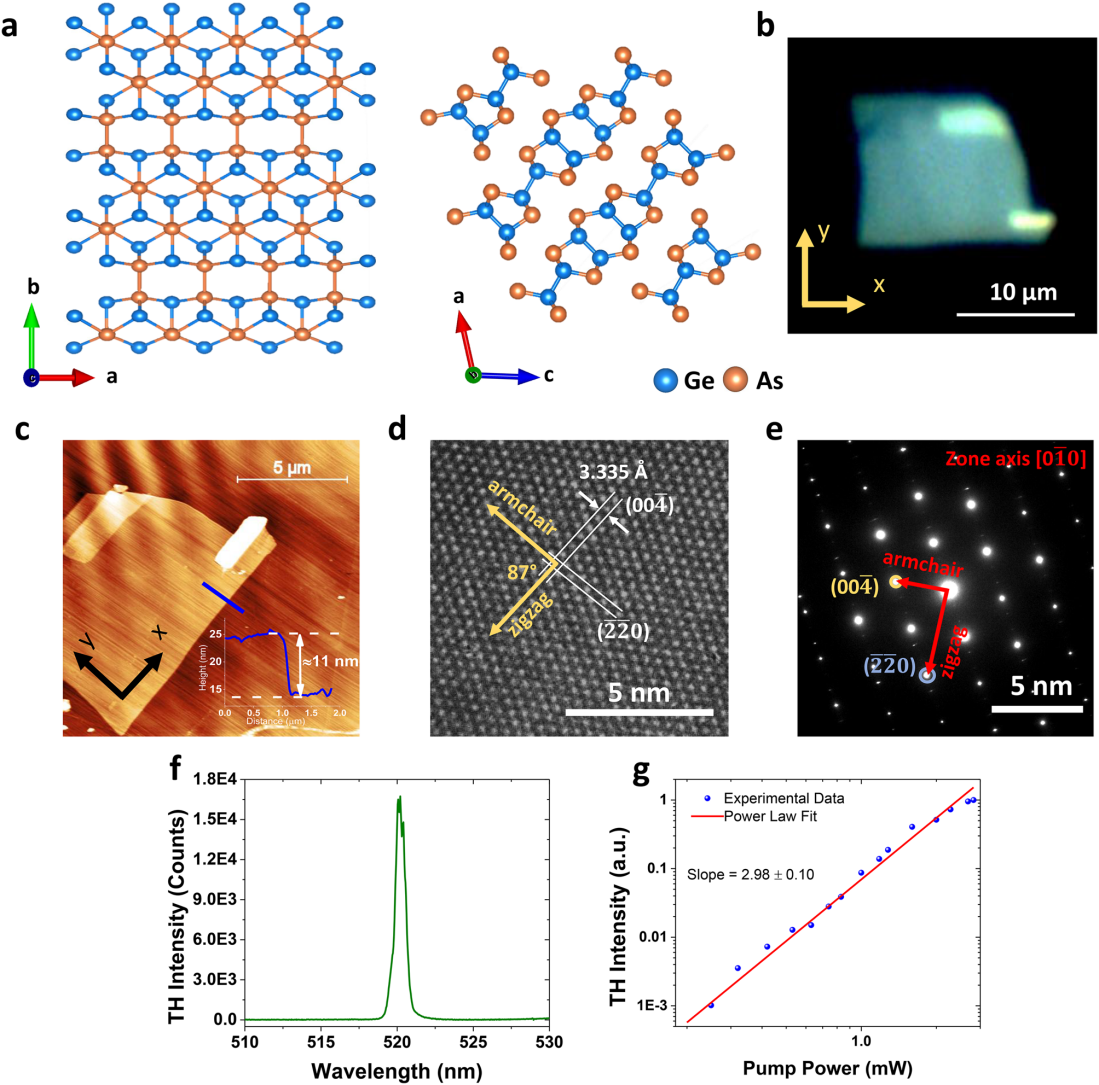
(**a**) Schematic representation of the top view and side view of the crystal structure of 2D layered GeAs. (**b**) Reflection microscopy image of the exfoliated GeAs flake. *x*- and *y*-axis in the inset mark the reference axes. (**c**) AFM image of the GeAs flake with the line profile showing the flake thickness of about 11 nm and the atomic smoothness. (**d**) HRTEM image of the GeAs flake. (**e**) Corresponding SAED pattern. (**f**) Optical spectrum of THG signal with a central wavelength of 520 nm, which is exactly one-third of the pump wavelength at 1,560 nm. (**g**) TH emission power as a function of the incident pump power.

The top view and side view of the layered GeAs crystal structure are illustrated in Fig. 1a. GeAs has a monoclinic crystal structure in the space group of C2/m (No. 12)^44^. The lattice of GeAs resembles that of GaTe with lattice parameters ***a*** = 3.833 Å, ***b*** = 8.453 Å, and ***c*** = 9.902 Å. The multilayer structure of this monoclinic crystal is formed by the stacked induvial layers along the *c*-axis by van der Waals forces, and the in-plane armchair and zigzag directions correspond to the *a*-axis and *b*-axis, respectively. This type of edge termination, corresponding to the formation of two different Ge–Ge bonds on different lattice directions, indicates the strong in-plane anisotropy nature of GeAs crystal^58^. The reflection microscopy image of one exfoliated 2D GeAs flake on quartz substrate is shown in Fig. 1b, where the *x* and *y* axes are assigned as the armchair and zigzag directions, respectively.

The surface flatness and thickness of the exfoliated GeAs flake are evaluated by the atomic force microscopy (AFM) measurement, and the flake thickness is extracted as approximately 11 nm from the height profile shown in Fig. 1c. To analyze the lattice structure of GeAs crystal, high-resolution transmission electron microscopy (HRTEM) and selected-area electron diffraction (SAED) measurements are performed on the transferred GeAs flake onto the Cu grid. The HRTEM image in Fig. 1d clearly shows the atoms as clear bright dots. The lattice spacing along [00$\overline{4}$] planes of 3.335 Å and an intersection angle of 87° between [00$\overline{4}$] and [$\overline{2}\overline{2}0$] planes are demonstrated in the HRTEM image. Figure 1e is the corresponding SAED pattern of HRTEM image and indicates a spot pattern which is consistent with a high-quality monoclinic structured single crystal of GeAs. The typical optical spectrum of THG signal measured with a spectrometer is plotted in Fig. 1f. As expected, the peak center of THG signal at 520 nm is exactly one-third of the pump wavelength at 1,560 nm. The THG process is confirmed by the measured TH emission power depending on the incident pump power, in which the experimental data is well fitted by the cubic power law with a slope of 2.98 ± 0.10, as shown in Fig. 1g.

The angle-resolved polarized Raman spectroscopy is a powerful technique commonly used to determine the crystal orientations^53,59–61^. Here, the angle-resolved polarized Raman spectroscopy in both parallel and perpendicular configurations is performed on the exfoliated GeAs flakes to label the crystal’s armchair and zigzag directions with the aid of HRTEM and SEAD measurements.

**Figure 2:**
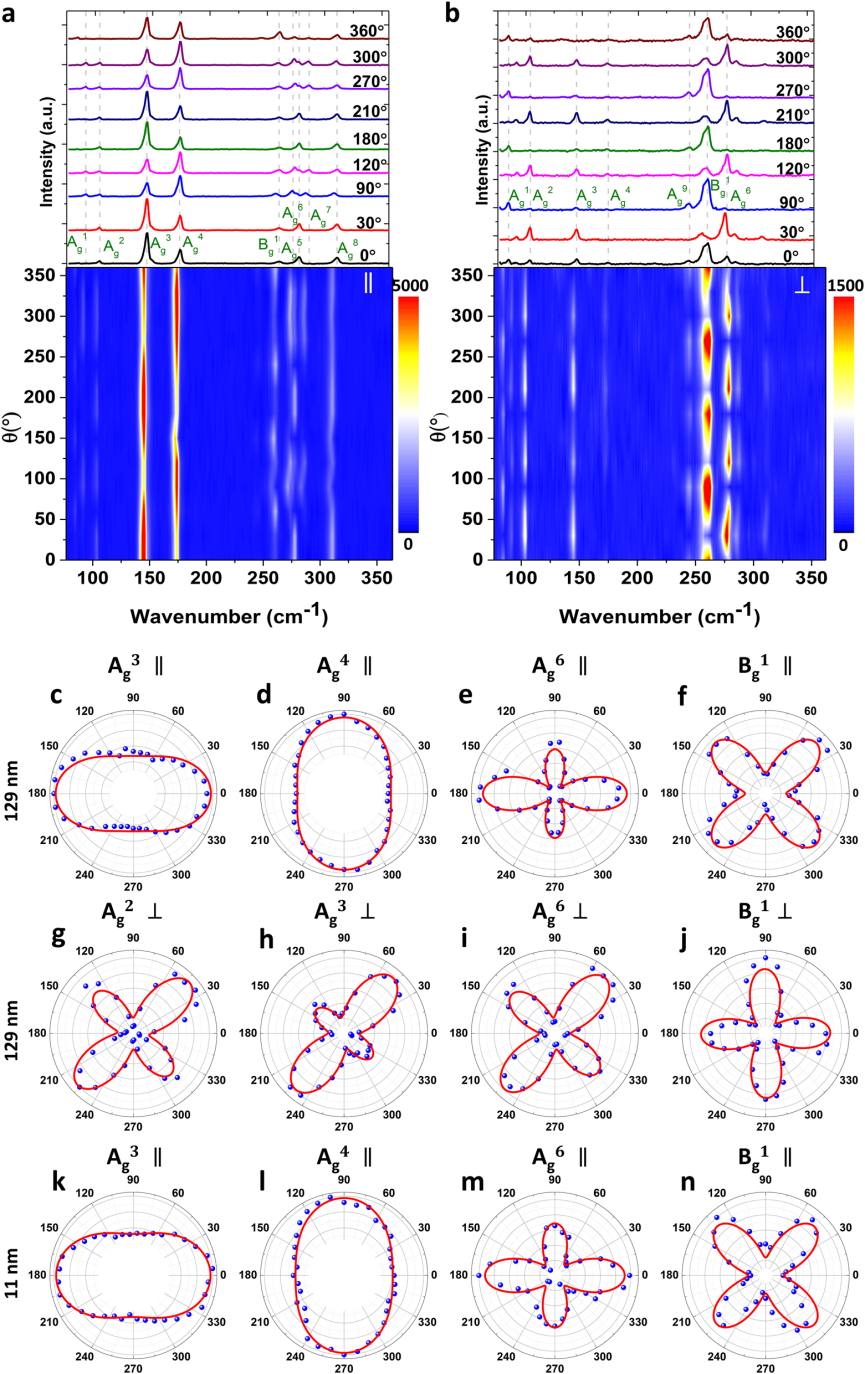
Angle-resolved polarized Raman spectra and the corresponding contour maps of the 129-nm thick GeAs flake acquired in (**a**) parallel and (**b**) perpendicular configurations. (**c**–**f**) Polar plots of the Raman intensities of $A_{g}^{3} \left( {147\;{\text{cm}}^{ - 1} } \right)$, $A_{g}^{4} \left( {174\;{\text{cm}}^{ - 1} } \right)$, $A_{g}^{6} \left( {276\;{\text{cm}}^{ - 1} } \right)$ and $B_{g}^{1} \left( {257\;{\text{cm}}^{ - 1} } \right)$ modes in parallel configuration for the 129-nm thick GeAs flake. (**g**–**j**) Polar plots for $A_{g}^{2} \left( {105\;{\text{cm}}^{ - 1} } \right)$, $A_{g}^{3} \left( {147\;{\text{cm}}^{ - 1} } \right)$, $A_{g}^{6} \left( {276\;{\text{cm}}^{ - 1} } \right)$ and $B_{g}^{1} \left( {257\;{\text{cm}}^{ - 1} } \right)$ modes in perpendicular configuration for the 129-nm thick GeAs flake. (**k**–**n**) Polar plots for $A_{g}^{3}$, $A_{g}^{4}$, $A_{g}^{6}$ and $B_{g}^{1}$ modes in parallel configuration for the 11 nm-thick GeAs flake. Blue dots are the experimental data and red solid curves are the fitted data with Eq. (1)–(4).

In the parallel and perpendicular configurations, the direction of linear polarization analyzer is set to be parallel and perpendicular to the excitation linear polarization direction, respectively. The GeAs flakes are fixed along *x* and *y* axes as shown in Fig. 1b. The linear polarization angle of the excitation beam from a 632.8 nm He–Ne laser is clockwise rotated around the *z*-axis from 0° (*x*-axis shown in Fig. 1b) to 360° with a step of 10° by using a half-wave plate. Figure 2a,b depicts the Raman spectra and the corresponding contour maps of the 129 nm-thick GeAs flake as a function of the linear polarization angle of the excitation beam under the parallel and perpendicular configurations. It is shown that some Raman active modes are observable in both configurations, but some others are available in only one configuration. Nine Raman active modes of the 2D GeAs flake are observed including eight $A_{g}$ modes and one $B_{g}$ mode^53^. The $A_{g}$ modes are at $94, 105, 147, 174, 271, 276, 283$ and 308 cm^−1^ which are assigned as $A_{g}^{1}$ to $A_{g}^{8}$, respectively, while the $B_{g}$ mode is at 257 cm^−1^ which is assigned as $B_{g}^{1}$. It is evident that the Raman mode intensities vary in a specific period with respect to the linear polarization angle of the excitation beam, with the Raman mode frequencies are almost unchanged. In order to further resolve of the Raman mode variation for the 129 nm-thick GeAs flake in detail, the polar plots of the four strongest Raman modes measured under parallel configuration (Fig. 2c–f for $A_{g}^{3}$, $A_{g}^{4}$, $A_{g}^{6}$ and $B_{g}^{1}$) and perpendicular configuration (Fig. 2g–j for $A_{g}^{2}$, $A_{g}^{3}$, $A_{g}^{6}$ and $B_{g}^{1}$) are depicted. The Raman intensities of $A_{g}$ modes show high anisotropy with a period of 180°. For the parallel configuration, the $A_{g}^{3}$ and $A_{g}^{6}$ modes reach the maximum intensities at 0° and 180°. In addition, the $A_{g}^{6}$ mode has a secondary maximum at $90^\circ$ and $270^\circ$. On the other hand, the $A_{g}^{4}$ mode shows a relatively weaker anisotropy with the maximum intensity at $90^\circ$ and $270^\circ$. Unlike the* A*_*g*_ modes, the $B_{g}^{1}$ mode exhibits a four-fold anisotropy with a period of $90^\circ$, having the maximum intensities along $45^\circ$ and $135^\circ$. The scenario for the perpendicular configuration is slightly different, where the $A_{g}^{2}$, $A_{g}^{3}$ and $A_{g}^{6}$ modes have the maximum intensities at $45^\circ$ and $225^\circ$ with a secondary maximum at $135^\circ$ and $315^\circ$. The maximum intensity for the $B_{g}^{1}$ mode occurs with a four-fold anisotropy along $0^\circ$ and $90^\circ$. Furthermore, to reveal the effect of the flake thickness on the anisotropy or crystal orientation, the Raman intensity variation of a 11 nm-thick GeAs flake under parallel configuration is also characterized (Fig. 2k–n for $A_{g}^{3}$, $A_{g}^{4}$, $A_{g}^{6}$ and $B_{g}^{1}$). It is found that the anisotropy pattern of the same Raman mode is almost unchanged for this really thin GeAs flake. Next, the experimental data is correlated with the theoretical Raman intensity functions for $A_{g}$ and $B_{g}$ modes. Considering $\overline{\overline{R}}$ as the Raman tensor of $A_{g}$ and $B_{g}$ modes and $g_{i}$ and $g_{s}$ as the unit polarization vectors of the incident and scattered light, the Raman intensity can be defined as $I\left( j \right) = \left| {g_{i} \cdot \overline{\overline{R}} \left( j \right) \cdot g_{s}^{T} } \right|$ where *j* is the mode number^45^. The Raman intensity of the $A_{g}$ modes in parallel and perpendicular configurations can be expressed as,

1 $$I^{||} \left( {A_{g} } \right) \propto \left( {cos^{2} \left( \theta \right) + \frac{a}{b} \cdot sin^{2} \left( \theta \right) \cdot \cos \left( {\phi_{ab} } \right)} \right)^{2} + sin^{4} \left( \theta \right) \cdot \cos^{2} \left( {\phi_{ab} } \right)$$

2 $$I^{ \bot } \left( {A_{g} } \right) \propto \left[ {\left( {cos^{2} \left( \theta \right) - \frac{a}{b}} \right)^{2} + \sin^{2} \left( {\phi_{ab} } \right)} \right] \cdot sin^{2} \left( \theta \right) \cdot \cos^{2} \left( {\phi_{ab} } \right)$$

The Raman intensity of the $B_{g}$ modes in parallel and perpendicular configurations are,

3 $$I^{||} \left( {B_{g} } \right) \propto \left| e \right|^{2} \cdot sin^{2} \left( \theta \right) \cdot cos^{2} \left( \theta \right)$$

4 $$I^{ \bot } \left( {B_{g} } \right) \propto e^{2} \cdot cos^{2} \left( {2\theta } \right)$$

where $a$, $b$, and $e$ are the constants of the Raman tensor, $\theta$ the linear polarization angle with respect to the crystal axis, and $\phi_{ab}$ represents the phase difference between *a* and *b.* It shows a good agreement between the experimental data (blue dots) and the fitted Raman intensity curves (red solid curves). Moreover, the anisotropy patterns of all Raman modes in both parallel and perpendicular configurations are consistent with the literature^53,54,62^. Hence, it is verified that *x*-axis ($0^\circ$) and *y*-axis ($90^\circ$) are identified as the armchair and zigzag orientations, respectively.

### Thickness-dependent in-plane anisotropic THG in exfoliated GeAs flakes

The strong anisotropic linear optical response of 2D GeAs foreshadows a similar anisotropic nonlinear response^54,58^. The expected anisotropic nonlinear responses of the GeAs flakes with various thicknesses are characterized by THG measurements. The linearly polarized pump beam at 1,560 nm with a spot size of $2.5\,\upmu {\text{m}}$ is used to excite the GeAs flake. A half-wave plate is placed before the sample and a linear polarization analyzer is placed in front of the spectrometer to extract the polarization dependence of the TH emission. The analyzer is fixed at either $0^\circ$ or $90^\circ$ for measuring the *x* and *y* components of TH emission while a half-wave plate is rotated in $5^\circ$ steps to change the linear polarization angle of the pump beam. The analytic expression of the emitted THG signal is derived by using the third-order nonlinear susceptibility tensor of the monoclinic GeAs crystal. The linearly polarized pump beam with the fundamental frequency $\omega$ can be defined as ${\mathbf{E}} = \left| {\mathbf{E}} \right|\hat{r}$ where $\hat{r} = \hat{x}\cos \theta + \hat{y}\sin \theta$, where $\theta$ is the polarization angle relative to the armchair (*x*-axis) direction of the crystal.

Knowing the monoclinic crystal structure of GeAs crystal with the space group of C2/m, the third-order nonlinear susceptibility tensor $\chi_{im}^{\left( 3 \right)}$ can be written as follows^63^:

5 $$\chi_{im}^{\left( 3 \right)} = \left[ {\begin{array}{*{20}c} {\chi_{11} } \\ 0 \\ {\chi_{31} } \\ \end{array} \begin{array}{*{20}c} 0 \\ {\chi_{22} } \\ 0 \\ \end{array} \begin{array}{*{20}c} {\chi_{13} } \\ 0 \\ {\chi_{33} } \\ \end{array} \begin{array}{*{20}c} 0 \\ {\chi_{24} } \\ 0 \\ \end{array} \begin{array}{*{20}c} {\chi_{15 } } \\ 0 \\ {\chi_{35} } \\ \end{array} \begin{array}{*{20}c} {\chi_{16} } \\ 0 \\ {\chi_{36} } \\ \end{array} \begin{array}{*{20}c} { \chi_{17} } \\ 0 \\ {\chi_{37} } \\ \end{array} \begin{array}{*{20}c} { \chi_{18} } \\ 0 \\ {\chi_{38} } \\ \end{array} \begin{array}{*{20}c} 0 \\ {\chi_{29} } \\ 0 \\ \end{array} \begin{array}{*{20}c} 0 \\ { \chi_{20} } \\ 0 \\ \end{array} } \right]$$

where $i$ equals to 1, 2, 3 which refers to $x,y,z$, respectively, and $m$ implies a combination of the three components as follows:

6 $$\begin{array}{*{20}c} {jkl} & {} & {xxx} & {yyy} & {zzz} & {yzz} & {yyz} & {xzz} & {xxz} & {xyy} & {xxy} & {xyz} \\ m & {} & 1 & 2 & 3 & 4 & 5 & 6 & 7 & 8 & 9 & 0 \\ \end{array}$$

Since, the excitation plane is the $x - y$ plane, the components with $z$ term can be set to zero. Hence, there will be only four non-zero terms left which results in the following TH electric field components:

7 $$E^{{\left( {3\omega } \right)}} = \left[ {\begin{array}{*{20}c} {E_{x}^{{\left( {3\omega } \right)}} } \\ {E_{y}^{{\left( {3\omega } \right)}} } \\ {E_{z}^{{\left( {3\omega } \right)}} } \\ \end{array} } \right] \propto \varepsilon_{0} E\left[ {\begin{array}{*{20}c} {\left( {\chi_{11} \cos^{3} \theta + 3\chi_{18} \cos \theta \sin^{2} \theta } \right)} \\ {\left( {\chi_{22} \sin^{3} \theta + 3\chi_{29} \sin \theta \cos^{2} \theta } \right)} \\ 0 \\ \end{array} } \right]$$

Then the generated TH intensity can be written as:

8 $$\begin{aligned} I_{x}^{{\left( {3\omega } \right)}} & = (E_{x}^{{\left( {3\omega } \right)}} )^{2} \propto \left( {\chi_{11} \cos^{3} \theta + 3\chi_{18} \cos \theta \sin^{2} \theta } \right)^{2} \\ I_{y}^{{\left( {3\omega } \right)}} & = (E_{y}^{{\left( {3\omega } \right)}} )^{2} \propto \left( {\chi_{22} \sin^{3} \theta + 3\chi_{29} \sin \theta \cos^{2} \theta } \right)^{2} \\ \end{aligned}$$

According to Eq. (7), the TH emission is also linearly polarized and the polarization angle is calculated as:

9 $$\theta_{THG} = \tan^{ - 1} \left( {\frac{{\chi_{22} \sin^{3} \theta + 3\chi_{29} \sin \theta \cos^{2} \theta }}{{\chi_{11} \cos^{3} \theta + 3\chi_{18} \cos \theta \sin^{2} \theta }}} \right)$$

**Figure 3:**
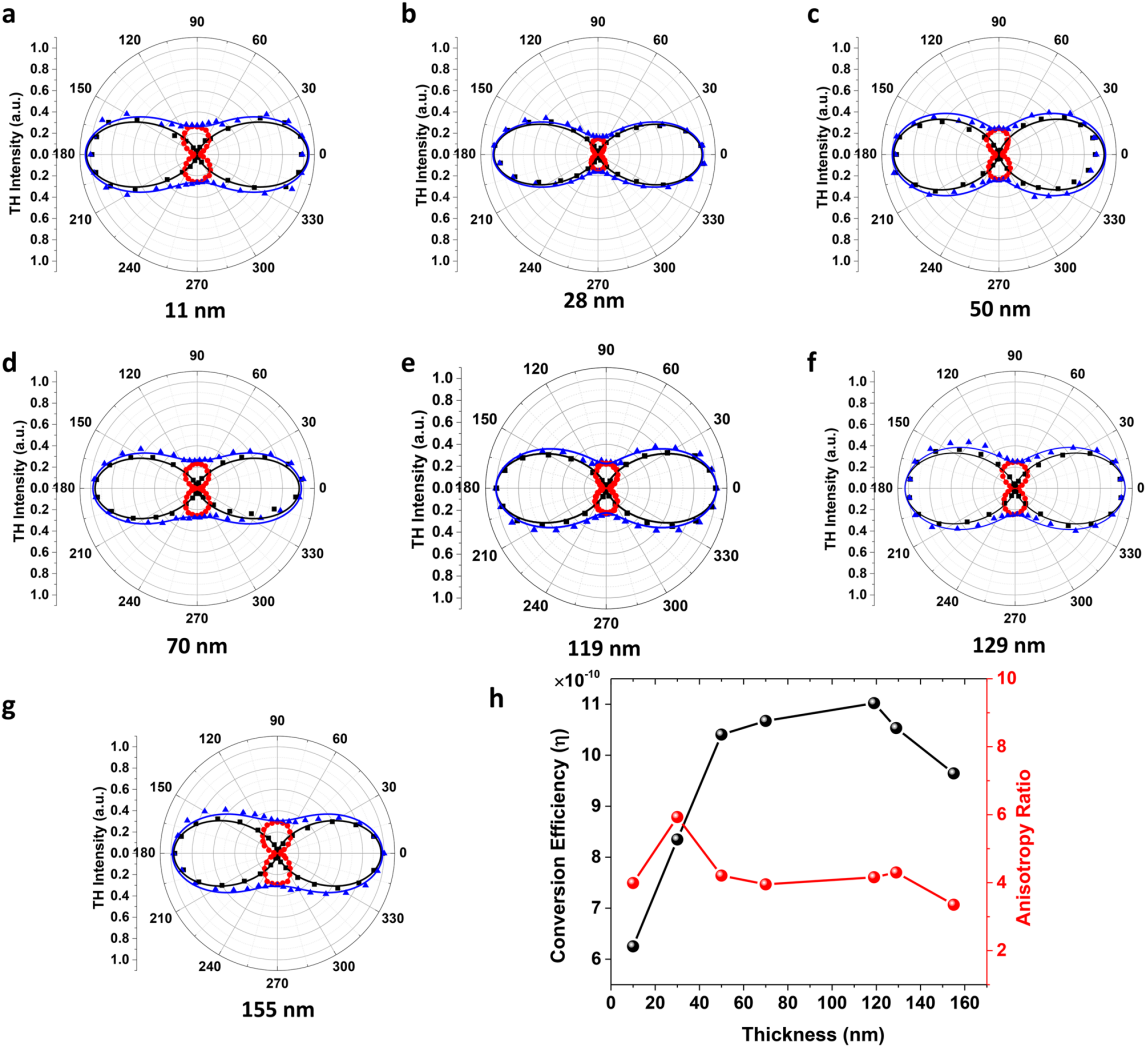
(**a**–**g**) Angular dependence of the THG intensity on the incident linear polarization for seven GeAs flakes with the thicknesses of 11 nm, 28 nm, 49 nm, 71 nm, 119 nm, 129 nm and 155 nm. Black squares, red circles and blue triangles refer to the measured *x*-component, *y*-component and total intensity of THG signal, respectively. Solid curves represent the corresponding theoretical fittings. (**h**) Evolution of the measured anisotropy ratio and THG conversion efficiency as a function of the flake thickness under the pump irradiance of 8.4 GW/cm^2^.

Figure 3a–g displays the angular dependence of the TH intensity on the incident linear polarization angle relative to the *x*-axis (armchair direction) for seven GeAs flakes with the thicknesses of 11 nm, 28 nm, 49 nm, 71 nm, 119 nm, 129 nm and 155 nm, showing the expected strong anisotropic nonlinear optical responses. The black squares and red circles represent the measured *x* and *y* components of TH emission $I_{x}^{{\left( {3\omega } \right)}}$ and $I_{y}^{{\left( {3\omega } \right)}}$, respectively, while the blue triangles correspond to the measured total TH emission $I^{{\left( {3\omega } \right)}}$. The maximum TH emission occurs as the incident linear polarization aligns to the *x*-axis (armchair direction) and the minimum TH emission is observed along the *y*-axis (zigzag direction). Furthermore, the solid curves in Fig. 3a–g are the fitted curves to the experimental data using Eq. (8), which indicates the good consistency between the theoretical fitting and the measured data. The observed anisotropy pattern is valid for the TH emission from all the GeAs flakes with different thicknesses. The $I_{x}^{{\left( {3\omega } \right)}}$ and $I_{y}^{{\left( {3\omega } \right)}}$ components of TH emission exhibit a two-fold polarization-dependent pattern for all the GeAs flakes. The TH anisotropy ratio is quantized as the ratio of the maximum TH intensity to the minimum one as $|I^{{\left( {3\omega } \right)}} |_{max} /|I^{{\left( {3\omega } \right)}} |_{min}$. Figure 3h plots the measured TH anisotropy ratio and THG conversion efficiency as a function of the flake thickness, and the corresponding values are also listed in Table 1.

**Table 1:** Comparison of the estimated relative magnitudes of the $\chi_{im}^{\left( 3 \right)}$ tensor components, the measured TH anisotropy ratio, and the measured THG conversion efficiency between the GeAs flakes with different thicknesses.

GeAs flake	Thickness (nm)	$\chi_{11}$	$\chi_{18}$	$\chi_{22}$	$\chi_{29}$	TH anisotropy ratio	Conversion efficiency (× 10^−10^)
#1	11	1.00	0.17	0.49	0.20	3.96	6.25
#2	29	1.00	0.16	0.39	0.18	5.93	8.35
#3	49	1.00	0.26	0.49	0.18	3.99	10.40
#4	71	1.00	0.17	0.49	0.20	3.84	10.70
#5	119	1.00	0.24	0.50	0.20	3.93	11.02
#6	129	1.00	0.22	0.47	0.18	4.30	10.53
#7	156	1.00	0.22	0.54	0.20	3.35	9.64

The anisotropy ratio of the THG in GeAs flakes varies from 3.35 to 4.30 for most flakes except for the 28 nm-thick flake with a higher ratio of 5.93. Such variations in nonlinear optical properties can be addressed to the introduced deformation or defects in the crystal lattice during the mechanical exfoliation process, which have also been observed previously for different kinds of nonlinear optical materials^37,45^. The relative magnitudes of the $\chi_{im}^{\left( 3 \right)}$ tensor components are estimated from the fitted curves using Eq. (8) and listed in Table 1. As expected, the estimated anisotropy between the components of $\chi_{im}^{\left( 3 \right)}$ tensor is consistent with the measured anisotropy ratio of TH emission. The THG conversion efficiency is measured with an average pump power of 1.5mW, which corresponds to an irradiance of 8.4 GW/cm^2^. The THG conversion efficiency increases from $6.25 \times 10^{ - 10}$ to $11.02 \times 10^{ - 10}$ as the flake thickness changes from 11 to 119 nm and then slightly decreases for thicker flakes. The optimal flake thickness range in terms of high THG conversion efficiency lies between 40 and 120 nm.

Furthermore, the observed THG properties from the monoclinic GeAs flakes are compared with those measured from the orthorhombic GeSe flakes^48^. The anisotropy ratios of THG in the orthorhombic GeSe flakes with the thicknesses of 93 nm and 163 nm are 2.77 and 4.37, respectively. That indicates the monoclinic GeAs flake with a similar thickness generally has a higher anisotropy ratio in the THG process. In addition, the THG conversion efficiency of the orthorhombic GeSe flake is in the range of $0.30 - 2.70 \times 10^{ - 9}$ for the thickness from 10 to 100 nm at 1 mW average pump power, but the monoclinic GeAs flake exhibits a lower THG conversion efficiency in the range of $0.20 - 0.35 \times 10^{ - 9}$ for the thickness from 11 to 120 nm at the same pump power. It is shown that the different crystal structures and lattice symmetries of 2D GeAs and GeSe have strong effects on the in-plane anisotropic nonlinear optical responses.

### Polarization state analysis of TH emission from the exfoliated GeAs flake

**Figure 4:**
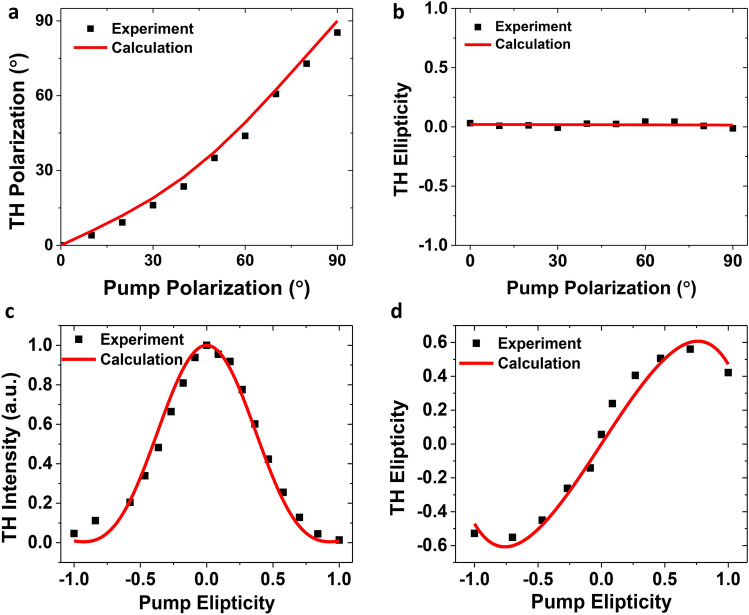
(**a**) Dependence of the TH polarization orientation on the pump polarization orientation. (**b**) Dependence of the TH ellipticity on the pump polarization orientation. (**c**) Variation of the TH intensity as a function of the pump ellipticity. (**d**) Variation of the TH ellipticity as a function of the pump ellipticity.

The polarization state of the emitted THG signal gives information on various aspects such as the spatial anisotropy of the crystal. Here, the polarization ellipticity $\varepsilon_{THG}$ and polarization orientation $\theta_{THG}$ of TH emission with respect to the pump polarization orientation $\theta$ for the 129 nm-thick GeAs flake is analyzed by measuring the Stokes parameters of TH emission as:

10 $$S_{0} = I^{{\left( {3\omega } \right)}} \left( {0^\circ } \right) + I^{{\left( {3\omega } \right)}} \left( {90^\circ } \right) = \left| {E_{x}^{{\left( {3\omega } \right)}} } \right|^{2} + \left| {E_{y}^{{\left( {3\omega } \right)}} } \right|^{2}$$

11 $$S_{1} = I^{{\left( {3\omega } \right)}} \left( {0^\circ } \right) - I^{{\left( {3\omega } \right)}} \left( {90^\circ } \right) = \left| {E_{x}^{{\left( {3\omega } \right)}} } \right|^{2} - \left| {E_{y}^{{\left( {3\omega } \right)}} } \right|^{2}$$

12 $$S_{2} = I^{{\left( {3\omega } \right)}} \left( {45^\circ } \right) - I^{{\left( {3\omega } \right)}} \left( {135^\circ } \right) = 2Re\left\{ {E_{x}^{{\left( {3\omega } \right)}} E_{y}^{{\left( {3\omega } \right)}} } \right\}$$

13 $$S_{3} = I_{RCP}^{{\left( {3\omega } \right)}} + I_{LCP}^{{\left( {3\omega } \right)}} = - 2Im\left\{ {E_{x}^{{\left( {3\omega } \right)}} E_{y}^{{\left( {3\omega } \right)}} } \right\}$$

where RCP and LCP refer to right- and left-handed circularly polarized light. Then the polarization ellipticity and polarization orientation can be expressed as $\varepsilon_{THG} = \tan \left( {\frac{1}{2}\sin^{ - 1} \frac{{S_{3} }}{{S_{0} }}} \right)$ and $\theta_{THG} = \frac{1}{2}\tan^{ - 1} \frac{{S_{2} }}{{S_{1} }}$, respectively. The dependence of the polarization orientation $\theta_{THG}$ of TH emission on the pump polarization orientation rotated from $0^\circ$ to $90^\circ$ is shown in Fig. 4a. The measured $\theta_{THG}$ (black squares) is consistent with the theoretical prediction (red solid curve) from Eq. (9). Furthermore, the ellipticity $\varepsilon_{THG}$ of TH emission with respect to the polarization orientation of pump beam is presented in Fig. 4b, which is almost zero for any incident linear polarization indicating the linearly polarized TH emission. In addition, the evolution of the output TH intensity and ellipticity is also investigated under the elliptically polarized pump beam. The analytic expression for the TH emission is derived in a similar way as the case of linearly polarized pump beam.

The elliptically polarized electric field of the pump beam with the major axis of the polarization ellipse oriented along the *x-*axis (armchair direction) can be defined as ${\varvec{E}} = \left| {\varvec{E}} \right|\hat{p}_{ \pm }$ where $\hat{p}_{ \pm } = \hat{x}\cos \varphi \pm \hat{y}i\sin \varphi$ with $\varphi$ being the ellipticity angle and the ellipticity calculated as $\varepsilon = \tan \varphi$. Then the TH electric field can be written as,

14 $$E^{{\left( {3\omega } \right)}} \propto \left( {\chi_{11} \cos^{3} \varphi - 3\chi_{18} \cos \varphi \sin^{2} \varphi } \right)\hat{x} + i\left( { - \chi_{22} \sin^{3} \varphi + 3\chi_{29} \sin \varphi \cos^{2} \varphi } \right)\hat{y}$$

The TH intensity is then written as,

15 $$I^{{\left( {3\omega } \right)}} \propto \left( {\chi_{11} \cos^{3} \varphi - 3\chi_{18} \cos \varphi \sin^{2} \varphi } \right)^{2} + \left( { - \chi_{22} \sin^{3} \varphi + 3\chi_{29} \sin \varphi \cos^{2} \varphi } \right)^{2}$$

Equation (14) indicates that the TH emission is elliptically polarized and the polarization ellipse has the same orientation as the pump beam. The ellipticity $\varepsilon_{THG}$ of TH emission is associated with the ellipticity $\varepsilon$ of pump beam by,

16 $$\varepsilon_{THG} = \frac{{ - \chi_{22} \varepsilon^{3} + 3\chi_{29} \varepsilon }}{{\chi_{11} - 3\chi_{18} \varepsilon^{2} }}$$

The elliptical polarization of pump beam is specified by a rotating quarter-wave plate (QWP) placed before the sample while setting the incident linear polarization along the *x*-axis (armchair direction). The ellipticity $\varepsilon$ of the input pump beam is gradually tuned from − 1 (RCP) to + 1 (LCP) by rotating the angle of the QWP fast axis ($\varphi$) with respect to the *x*-axis. Figure 4c plots the variation of TH intensity as a function of the ellipticity of pump beam. The TH intensity is maximum for $\varepsilon = 0$ which refers to the linearly polarized pump beam along the armchair direction of GeAs crystal. Moreover, the TH intensity decreases to almost zero as the pump beam polarization is changed from linear to circular polarization with $\varepsilon$ tuned from 0 to 1 or − 1. Figure 4d plots the ellipticity $\varepsilon_{THG}$ of TH emission obtained from the measured Stoke parameters as a function of the pump ellipticity $\varepsilon$, which is consistent with the theoretical prediction from Eq. (16).

## Discussion

In summary, we have observed strong in-plane anisotropic THG in exfoliated GeAs flakes with different thicknesses. The angle-resolved polarized Raman spectroscopy and HRTEM measurements are used to determine the armchair and zigzag orientations of the GeAs crystal. Highly anisotropic third-order nonlinear optical response with respect to the incident linear polarization of pump beam has been demonstrated by correlating the experimental data with the theoretical prediction. The dependence of THG conversion efficiency on the flake thickness is explored to reveal the optimal thickness range for high conversion efficiency. The TH anisotropy ratio for the GeAs flakes is also measured and the averaged anisotropy ratio around 4 is obtained. In addition, the components of anisotropic third-order nonlinear susceptibility tensor of the GeAs flakes are also estimated from the experimental data. Furthermore, the polarization state analysis of TH emission is performed for linearly and elliptically polarized incident pump beam, showing that the intensity and polarization state of TH emission can be manipulated by the incident polarization of pump beam. These first results on the nonlinear optical properties of the newly introduced layered group IV-V compound GeAs will shed light on future nonlinear photonic applications in photonic and quantum chips, optical communication and optical information processing.

## Methods

### Sample preparation

2D GeAs flakes are mechanically exfoliated from a bulk GeAs single crystal (2D Semiconductors) using typical scotch tape method onto the pre-cleaned quartz substrate. Before the transfer of the exfoliated GeAs flakes, the quartz substrate is sonicated in acetone, isopropanol alcohol and deionized water, respectively. For the HRTEM measurement, thin GeAs flakes are transferred on the Cu grid with a polydimethylsiloxane (PDMS) film.

### Optical setup

The linearly polarized laser beam at 1560 nm (Calmer fiber laser, pulse width 90 fs, repetition rate 80 MHz) is prepared by a linear polarizer and a half-wave plate, and then focused on the GeAs flake by a 20 × objective lens with *NA* = 0.40. The TH emission is collected by a 50 × objective with *NA* = 0.42, transmitted through a shortpass filter to block the pump beam, and coupled into a spectrometer (Horiba, iHR520). For preparing the elliptically-polarized pump beam, instead of the half-wave plate, a quarter-wave plate is rotated before the GeAs flake. For measuring the Stokes parameters of the TH emission, another set of a quarter-wave plate and a linear polarizer was mounted before the spectrometer. For the angle-resolved polarized Raman spectrum measurements, a 632.8 nm He–Ne laser beam is transmitted through a linear polarizer and a half-wave plate, then focused on the GeAs flake by a 60 × objective lens with *NA* = 0.85. The back-reflected Raman signal is passed through a Rayleigh rejection filter (Semrock, LP02-633RE-25) and collected into the spectrometer. The parallel and perpendicular polarization components of the Raman spectrum are measured by using linear polarization analyzer just before the spectrometer.
